# Non-reducing End of Heparin Tri-saccharide is a Scavenger Tool to Detoxify the Glucose Toxicity in Diabetes

**DOI:** 10.33696/immunology.5.171

**Published:** 2023

**Authors:** Andrew Jun Wang, Aimin Wang, Vincent Charles Hascall

**Affiliations:** 1 School of Medicine, New York Medical College, Valhalla, NY 10595, USA; 2 Department of Biomedical Engineering, Lerner Research Institute, Cleveland Clinic, Cleveland, OH 44195, USA

**Keywords:** Hyperglycemia, Intracellular hyaluronan, ER stress, Autophagy, Hyaluronan matrix, Inflammation, Mesangial cell, Diabetic nephropathy, Heparin, Heparin trisaccharide

## Abstract

Heparin is a highly sulfated, hence highly polyanionic, glycosaminoglycan with a repeating disaccharide that contains a hexuronic acid, and it has been used as an anticoagulant clinically for more than half a century. Daily IP injections of small amounts of heparin in the STZ diabetic rat prevented these pathological responses even though the animals sustained hyperglycemic levels of glucose throughout. However, the structural determinant that mediates this activity is not clear. This paper describes our finding that the responses of hyperglycemic dividing mesangial cells to heparin are mediated by its non-reducing terminal trisaccharide and proposes that the non-reducing end tri-saccharide of heparin acts as a scavenger tool to detoxify the glucose toxicity in diabetes.

## Introduction

Mesangial expansion is the principal glomerular lesion in DN that reduces the area for filtration and leads eventually to sclerosis and renal failure [1,2]. However, the mesangial extracellular matrix expansion and sclerosis are preceded by an early phenotypic activation and proliferation of the glomerular mesangial cells [3] that is followed by a prominent glomerular infiltration of monocytes and macrophages [3,4] that associate with glomerular hyaluronan (HA) extracellular matrix formation. Glomerular monocytes and macrophages have been prominently identified in DN in both animal models [4] and humans [5], and appear to have a key role in the induction of mesangial matrix expansion by elevating glomerular TGF-β [6–8], hypercellularity, and the onset of proteinuria [9,10], which are characterized by inflammatory processes [11,12].

Daily IP injections of a small amount of heparin in the STZ diabetic rat prevented these pathological responses even though the animals sustained hyperglycemic levels of glucose throughout the experiments [13,14]. This led to clinical trials with heparin for treatment of diabetic patients [15]. However, the molecular and cellular mechanism(s) underlying the roles of heparin are still unclear. Thus, we studied the roles of heparin in regulating the hyperglycemia induced responses in dividing cells.

## Heparin and Its Non-anticoagulant Roles

Heparin is a highly sulfated, hence highly polyanionic, glycosaminoglycan with a repeating disaccharide that contains a hexuronic acid (either glucuronic acid or iduronic acid) and glucosamine (either N-acetylated or N-sulfated). It is synthesized in mast cells as serglycin, a proteoglycan that contains 15–20 heparin chains with MWs of ~120 kDa. During or after entry into the secretory granules the chains are hydrolyzed by heparanase to 10–15 kDa [16,17]. Clowes and Karnovsky [18] in 1977 showed that the antimitogenic and/or antiproliferativeactivities of heparin in smooth muscle cells could be independent of its well-known anticoagulant activity. Since then, continuous efforts have been made that showed that heparin inhibits mesangial cell growth in both experimental renal disease models [13,14,19–21] and in cell culture experiments [22,23]. Our studies provide evidence that heparin interacts with a receptor on hyperglycemic dividing cells that blocks glucose uptake within 1 hour after entering G1, which prevents the intracellular accumulation of HA during division in hyperglycemia [24–26]. Further, the cells are then reprogrammed to synthesize an extensive extracellular HA matrix after division by HAS enzymes in the cell membrane to address the ongoing glucose stress. This new HA matrix binds M2 macrophages, which are anti-inflammatory, and they activate to digest and remove the HA matrix. The impact of this heparin induced process prevents the intracellular stress responses by maintaining lower levels of cytosolic UDP-sugars [16,25,27].

Our previous studies [28–31] support our proposed model that heparin binds to a receptor on the surface of quiescent, growth arrested G0/G1 RMCs that allows the cells to inhibit hyperglycemia-induced responses during cell division based on the following observations. 1) Binding of heparin to RMCs is specific, rapid (5–10 min), saturable (within 60 min) and reversible. 2) Scatchard analysis of heparin binding indicates a single class of receptors identified as calreticulin [29,31] with ~6.6 × 10^6^ binding sites per cell (K_d_ = 16 nM), which is similar to the K_d_ of 20 nM observed in quiescent cells from different assays in our previous publication [26,29]. 3) Surface-bound heparin can be internalized and degraded [26,29]. 4) The affinities and numbers of heparin binding sites are affected by the stage of RMC growth. and 5) Heparin acts on the RMC surface to affect both PKC-dependent and independent pathways [29]. These results indicate: 1) that exposure of RMCs to heparin in the early stage of the cell cycle is sufficient to induce the internalization of heparin into the peri-nuclear region and then into the nucleus [29,31]; and 2) that calreticulin, a component of cell surface complexes, is the cell surface protein on the mesangial cell surface mediating interaction of heparin to mesangial cells, which can initiate signals to regulate the molecular and cellular responses of RMCs dividing under hyperglycemic glucose.

## Non-reducing Trisaccharide of Heparin

There is only one heparanase in the human genome, and it hydrolyzes bonds between hexuronate residues and GlcNS(6S) on heparin or heparan sulfate [16,17]. Previous studies showed that mesangial cells in normal glucose catabolize cell surface heparan sulfate proteoglycans by an endosomal pathway that contains heparanase, which hydrolyzes the heparan sulfate chains (50 kDa) into fragments (5–10 kDa), and that a large proportion is recycled into the medium [28,32]. These fragments bound to the surface of G0/G1 growth-arrested cells in normal glucose, but not to confluent cells, and inhibited serum-stimulated mitogenesis of mesangial cells. Further, the intact heparan sulfate chains isolated from the cell surface proteoglycans showed much less activity. Neither trypsin treatment, heating to 90° C for 20 min, nor chondroitinase digestion had an effect on the ability of the heparan sulfate fragments to suppress thymidine incorporation in the stimulated cells, whereas nitrous acid degradation did. This supports the interpretation that the activity is a property of the heparan sulfate fragments themselves. The degradation of intact heparan sulfate chains by heparanase occurs by hydrolyzing bonds between hexuronate residues and GlcNS(6S) in the interspaced highly sulfated regions typical of heparan sulfate [28,32]. Therefore, the heparan sulfate fragments contain the newly formed non-reducing ends with GlcNS(6S), which provides evidence that the highly sulfated non-reducing end with this motif is also critical for their binding activity to dividing mesangial cells.

Further, our studies were undertaken to determine whether the non-reducing end of heparin is sufficient to mediate the roles of intact heparin in inducing monocyte adhesive hyaluronan matrix formation [26]. Bacterial heparin lyase is an endo-eliminase that converts heparin primarily to disaccharides containing unsaturated (Δ-hexuronate) residues at their non-reducing ends [26]. When heparin lyase acts it also forms a saturated trisaccharide (Hep Digest/Tri) from the non-reducing end of the heparin chain formed through the action of heparanase on serglycin [16,17,26]. The purified Hep Digest/Tri sample contains only a small amount of the Δ-Di. Thus, the mass of the Hep Digest/Tri used in the following experiment was <1/10 that of the heparin, i.e. they were at equivalent molar concentrations. The purified non-reducing trisaccharide of heparin (Hep-Tri) at 1/10 the concentration (~20 nM) was as effective as heparin in increasing HA synthesis in hyperglycemic dividing RMCs [26]. Hep-Tri does not have anti-coagulant activity and does not bind to a variety of potentially important growth factors [33,34], which have been complicating factors for the use of heparin in the diabetic rat model and in other translational studies that have been tried. Thus, future studies need to focus on the role of Hep-Tri in vivo.

## Future Studies

The beneficial effects of heparin and its derivatives in DN have been extensively explored in both experimental animal models and clinical trials [13–15]. The outcomes are varied, depending on doses, period of treatments, and the batches used. A recent meta-analysis showed a renoprotective benefit of heparin in patients with diabetes and micro- and macroalbuminuria [35]. However, the mechanism(s) are still unclear. Particularly pertinent to our studies, a recent study concluded that unfractionated heparin (UFH) and the different low MW heparins (LMWHs) exert specific effects on podocyte permeability, and they underline the need of in vivo tests to evaluate new biological non-anticoagulant properties of LMWH [36]. Thus, a future study is needed to determine the roles of heparin/Hep-Tri in detoxifying the glucose toxicity resulting from hyperglycemia.

**Figure 1.**
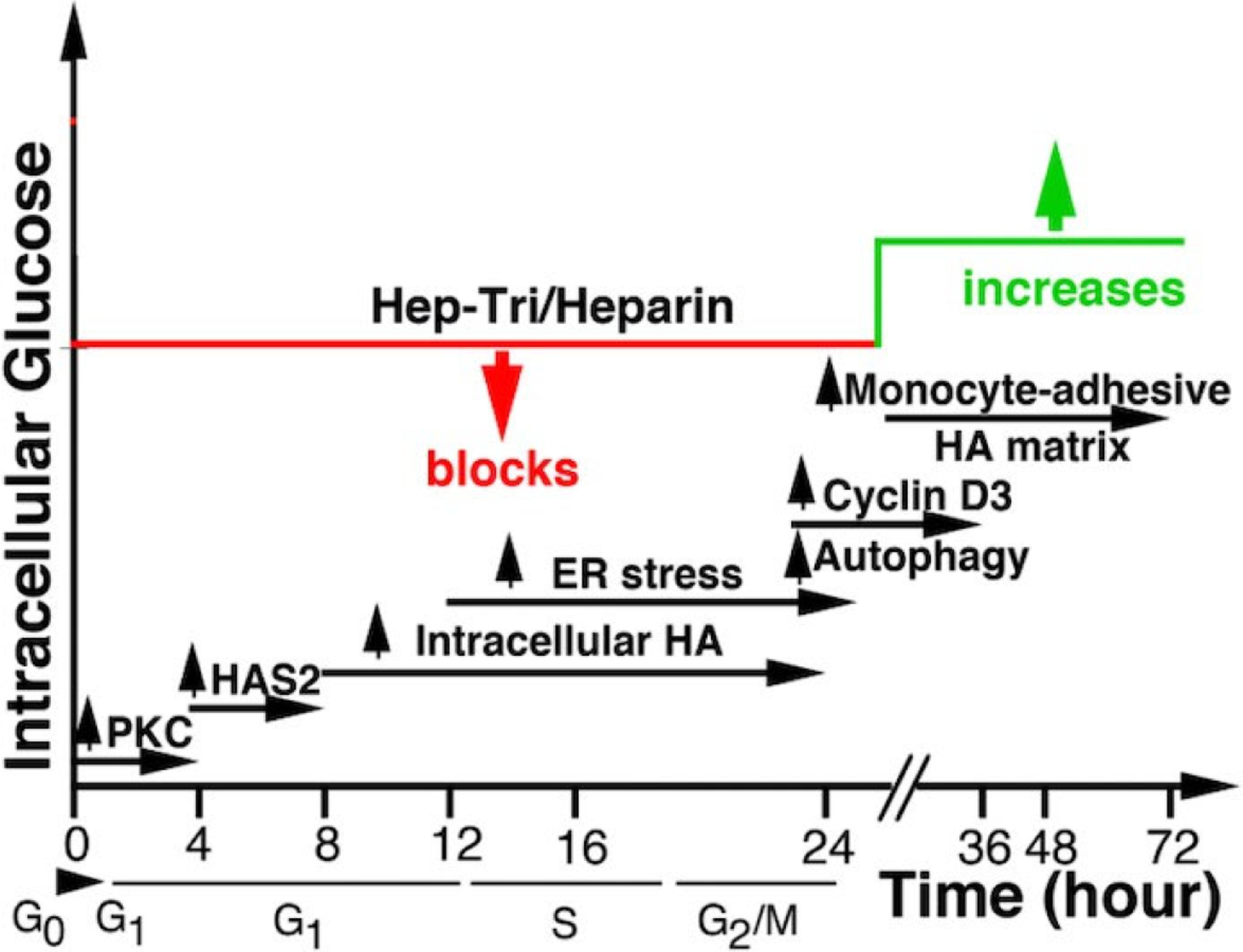
Proposed timeline events regulated by heparin/Hep-Tri in hyperglycemic dividing RMCs.

Our model (Figure 1) for the hyperglycemia induced abnormal responses in mesangial cells entering G1 division contains the following cellular and molecular timeline events: **1**) continuous uptake of glucose in high glucose conditions (0–4 hrs); **2**) activation of PKC signaling (0–4 hrs); **3**) activation of the hexosamine biosynthesis pathway with increased UDP-GlcNAc (6–12 hrs); **4)** activation of HAS2 in intracellular compartments (10–16 hrs) that abnormally synthesize HA into ER, golgi and transport vesicles (10–24 hrs); **5**) ER stress and autophagic responses (12–24 hrs); **6**) upregulation of cyclin D3 (24–30 hrs); **7**) extrusion of HA to form the abnormal extracellular monocyte-adhesive HA matrix after division (30–48 hrs); and **8**) activation of pro-inflammatory responses (30–48 hrs); which leads to development and progression of DN.

Our model for the effects of heparin/Hep-Tri is: **1)** Heparin/Hep-Tri interacts with a receptor on dividing RMCs. **2)** This binding initiate intracellular signaling pathways that block glucose transport by removing Glut4 from the cell surface within 1 hour from G0/G1 [25,31]. **3)** On completion of division, the high glucose influx from the sustained hyperglycemia is now addressed by activating HA synthesis at the plasma membrane. **4)** Subsequent synthesis and extrusion of the monocyte-adhesive HA matrix outside the cell maintains cytosolic UDP-GlcNAc within a normal concentration, thereby sustaining RMC and podocyte functions. **5)** Further, within glomeruli, influxed anti-inflammatory tissue repair M2 macrophages remove the HA matrix, which allows sustained kidney glomerular function.

Our studies have shown: **1**) that glomeruli isolated from 4–6 week STZ diabetic rats contain 3–5 times as much HA as glomeruli from control rats; and **2**) that glomeruli from heparin treated STZ diabetic rats have 3–5 times as much HA at 1–2 weeks, which decreases to near control level at 6 weeks [12,24,37]. Sections of 6 week kidneys: **1)** showed extensive infiltration of macrophages in glomeruli of both heparin treated and untreated STZ diabetic rats; and **2**) showed the presence of cyclin D3 and LC3 in the glomeruli of the STZ diabetic rat but not in the heparin treated STZ diabetic rat [12,24,37]. These results indicate that similar processes to those observed in RMC cultures are likely occurring in vivo. Thus, objectives in the future studies will be: **1**) to delineate these molecular and cellular events in the course of development and progression of DN using STZ-induced diabetic rats, and **2**) to determine the effects of treatment of STZ diabetic rats with the Hep-Tri on inhibition of DN progression. Further, the use of Hep-Tri can avoid the unsuccessful clinical attempts with undefined low MW heparin [38,39]. These studies will provide the evidence for the extent that Hep-Tri attenuates the progression of the disease by preventing the intracellular HA network formation.
